# Distribution of Virulence Factors in Vancomycin-Resistant Enterococci Isolated from Clinical and Intestinal Samples

**DOI:** 10.3390/microorganisms14010090

**Published:** 2025-12-31

**Authors:** Preslava Mihaylova Hristova

**Affiliations:** Department of Microbiology and Virology, Faculty of Pharmacy, Medical University-Pleven, 1st St. Kliment Ohridski Str., 5800 Pleven, Bulgaria; preslavahristova@outlook.com; Tel.: +359-64-884-152

**Keywords:** vancomycin-resistant enterococci, *acm* gene, *esp* gene

## Abstract

Virulence factors in enterococci play an important role in the pathogenesis of enterococcal infection and colonization. The aim was to determine the prevalence of genes encoding virulence factors in VRE from clinical and intestinal samples. A total of 163 VRE (94 clinical and 69 intestinal) isolated from patients treated in the University Hospital were studied. Species identification was performed by Vitek 2. The genes for vancomycin resistance (*vanABCDMN*) and virulence factors (*ace*/*acm*, *asa1*, *esp*, *efaA*, *cylA*, *gelE* and *hyl*) were detected by multiplex PCR. The prevalence of virulence genes with respect to clinical and intestinal *E. faecium* was compared using Fisher’s exact test and *p* > 0.05 was considered statistically significant. Carriers of virulence factors were 107 VRE: 85 clinical and 14 intestinal *E. faecium*, 6 intestinal *E. gallinarum* and single *E. durans* and *E. faecalis*. The dominant virulence genes were *acm* and *esp*. Genes for virulence factors were not detected in the tested *E. casseliflavus* isolates. There was no statistically significant difference in the prevalence of the genes encoding virulence determinants between the clinical and intestinal *E. faecium*. High diversity of virulence determinants was found in 107 VRE and a combination of two genes, mainly *acm* and *esp*, was detected in 94 of them.

## 1. Introduction

Enterococci are part of the normal microbiota of the human intestinal tract. Also, these bacteria are causative agents of a wide spectrum of nosocomial infections such as urinary tract infections, intra-abdominal, pelvic and postoperative infections, bacteremia, and infective endocarditis. In rare cases, they have been associated with the pathogenesis of central nervous system infections, respiratory tract infections, eye infections, etc. [1,2].

Enterococci are widely spread worldwide. They are the second-most common Gram-positive bacteria in United States, Europe, and China associated with hospital-acquired infections (HAIs) [3,4,5]. Significant healthcare challenge are vancomycin-resistant enterococci (VRE), particularly vancomycin-resistant (VR) *E. faecium* [6]. In Australia, the Australian Passive AMR Surveillance Report for 2023 concluded that 42.5% of *E. faecium* isolates are resistant to vancomycin [7]. The latest ECDC Annual Epidemiological Report for 2024 [8] shows that the average percentage of invasive VR *E. faecium* is 16.5% and it ranged from 0.0% in Iceland and Luxemburg to 61.7% in Lithuania. In Bulgaria, until 2012, the incidence of invasive VR *E. faecium* was 0%. After that, a trend towards an increase was noted and in 2024, the national percentage of VR *E. faecium* for Bulgaria was 14.5% [9]. All these data testify for the rapid spread of VRE in the hospitals.

The pathogenicity of enterococci is associated with their ability to adhere to the epithelium of the urinary tract, oral cavity, and embryonic kidney cells; to attach to extracellular matrix proteins and inert materials such as a number of medical devices; and to evade the immune system and form a biofilm, making them resistant to antibiotic action and phagocytic attack [10,11]. The capability of enterococci to acquire new traits contributes to virulence, allowing them to colonize new areas in the host and to cause different types of infections [12].

The virulence of enterococci is regulated by genes located in specific regions of the cell genome, called “pathogenicity islands” (PAIs) [13]. The first report for the presence of PAIs in the genome of a multidrug-resistant nosocomial *E. faecalis* strain (MMH594) was described by Huycke et al. in 1991 [14]. The genomic region was about 150 kb in size and contained genes encoding transposases, transcriptional regulators, and proteins with a role in the virulence. The PAI genes of *E. faecalis* were subsequently found to be responsible for the production of enterococcal surface protein, secretory cytolysin, and aggregation substance [15,16]. Usually, PAIs are closely related to virulent enterococcal clones and are often modified or even absent in less virulent strains. The changes that occur in the enterococcal PAIs are an important element for their evolution [12,17].

Virulence factors in enterococci can be divided into three main groups: surface adhesion factors, hydrolytic enzymes, and secreted virulence factors [10]. The first group includes pili, aggregation substance, and extracellular surface proteins. Hydrolytic enzymes include hyaluronidase, gelatinase, and serine protease. A major secreted virulence factor is the cytolysin, also known as β-hemolysin.

The enterococcal surface protein (Esp) is found in the cell wall of *E. faecium* and *E. faecalis* and its production is encoded by the *esp* gene. In vitro conjugative transfer of the *esp* gene has been demonstrated in the two strains. Esp is widely distributed among different strains of *E. faecalis* whereas, in *E. faecium*, it is predominantly presented in hospital-acquired isolates [18]. The functions of this virulence factor is to promote the adhesion, colonization, and evasion of immune system defense mechanisms [19]. Heikens et al. [20] found that *E. faecium* Esp is responsible for biofilm formation and plays a role in the pathogenesis of endocarditis and bacteremia. Another virulence factor is the aggregation substance (AS) found in *E. faecalis* and encoded by conjugated plasmid (*asa1* or *aggA*). AS enhances the adhesion to renal tubular cells [21] and promotes the attachment and survival of enterococci in macrophages [22]. Collagen-binding proteins play a key role in the pathogenesis of endocarditis as they help for the attachment of enterococci to tissue cells [23]. Adhesion to collagen in *E. faecalis* (Ace) is encoded by the *ace* gene and the adhesion to collagen of *E. faecium* (Acm) is encoded by the *acm* gene. It is interesting to note that the functional *acm* gene is present mainly in clinical isolates [24]. EfaA (*E. faecalis* antigen A) is a major cell surface antigen in *E. faecalis* identified using sera from patients with endocarditis. It is encoded by the *efaA* gene. Data show that the *efaA* gene is present in almost all *E. faecalis* isolates, and its homolog has also been found in *E. faecium* [25]. Gelatinase is an extracellular Zn-containing metalloproteinase that hydrolyzes gelatin, collagen, and other proteins. The gene encoding the production of gelatinase (*gelE*) is located on an operon together with the *fsrE* gene encoding the serine protease [26]. Mutations in the *fsr* operon and inactivation of the *fsr*-control gene *gelE* indicate the important role of gelatinase in biofilm formation in *E. faecalis* [27]. Hyaluronidase (Hyl) produced by *E. faecalis* and *E. faecium* is a hydrolytic enzyme that degrades hyaluronic acid with subsequent tissue invasion and provides nutrients to bacteria. Its synthesis is encoded by the *hyl* gene which is carried on a plasmid. Gomez et al. [28] described a megaplasmid (*hylEfm*) in *E. faecium* containing the *hyl* gene, antibiotic resistance genes, and metabolic genes. These megaplasmids are widely spread among clinical *E. faecium* isolates and play a key role in colonization. Cytolysin (Cyl), also called β-hemolysin or bacteriocin, is encoded by the *cyl* gene in *E. faecalis*. The Cyl has lytic activity against various types of eukaryotic cells, including immune cells [29,30]. Several studies have testified for the role of *E. faecalis* Cyl in the pathogenesis of enterococcal infection. Huycke et al. [14] found that the mortality rate in patients with bacteremia caused by hemolytic, gentamicin-resistant strains of *E. faecalis* was five times higher than in patients infected with nonhemolytic, gentamicin-susceptible strains.

The number of studies investigating the distribution of virulence factors among non-*faecalis*/non-*faecium* enterococcal species is limited [31,32,33]. In a study from 2016, the most common gene detected in 5/33 *E. mundtii*, 4/35 *E. raffinosus*, 3/9 *E. solitaries*, 2/20 *E. malodoratus*, 2/10 *E. dispar*, and 1/17 *E. hirae* was *gelE*. Additionally, *cylA*, *hyl* and *asa1* were confirmed in a single vancomycin-susceptible (VS) non-*faecalis*/non-*faecium* strain [31]. Sienko et al. [32] detected *esp*, *hyl*, and *acm* in six unusual vs. enterococci (five *E. avium* and one *E. durans*).

The type and number of virulence factors in VR *E. faecalis* and *E. faecium* varies among different studies [34,35,36,37]. Haghi et al. [37] investigated the frequency of genes encoding virulence factors in 79 VRE (69 *E. faecalis* and 10 *E. faecium*) isolated from urine and found *esp* in 67.1% of the strains, followed by *PAI* (45.5%) and *sprE* (41.7%). Carriage of two or more genes was detected in 67 (97.1%) *E. faecalis* and in 5 (50%) *E. faecium*. Iranian authors [36] found that, among 190 VR *E. faecalis* and 75 VR *E. faecium*, the most identifiable virulence gene was *asa1*. In addition, *esp* and *hyl* genes have also been demonstrated both in *E. faecalis* and *E. faecium*. Jovanovic et al. [34] investigated the prevalence of *esp* and *hyl* among VRE isolates from five hospitals in Belgrade, Serbia. The authors confirmed these genes in 45 (29.2%) and 43 (27.9%) *E. faecium* isolates and in 16 (76.2%) and 0 (0%) *E. faecalis* isolates, respectively. Only in eight *E. faecium* were the *esp* and *hyl* in combination.

Regarding the clinical significance of VRE, the wide diversity of virulence determinants among VR *E. faecium* and VR *E. faecalis* and the limited amount of scientific data about the distribution of virulence genes among VR non-*faecalis*/non-*faecium* species, the aim of the current study was to determine the prevalence of genes encoding virulence factors in VRE species isolated from the clinical and intestinal samples of hospitalized patients.

## 2. Materials and Methods

### 2.1. Bacterial Isolates

The present study was performed on 163 non-repeated VRE isolated from patients treated in the University Hospital, “Dr. G. Stranski”, Pleven, Bulgaria from January 2016 to December 2020. The research was approved by the local ethics board of Medical University-Pleven. Data processing was anonymized and complied with local data protection legislation (No. 455/21.06.2017 and No. 512/03.05.2018) and with the European Directive on the Privacy of Data (95/46/EC). All subjects that participated in this study gave a written informed consent in accordance with the Declaration of Helsinki.

Various samples were obtained from symptomatic or suspected for colonization patients using sterile swabs, containers, or BD BACTEC™ blood culture bottles (Becton Dickinson, Wokingham, UK). They were directly transported to the Department of Clinical Microbiology with subsequent culturing on the appropriate culture media. All samples obtained from the patients, over 18 years, suspected for infection or colonization in our hospital were included in the study. Duplicated enterococci isolated from the same patient or the same samples were excluded from the analysis. Each enterococcal isolate carrying any of the *van* genes of resistance were defined as VRE.

A total of 94 VRE were the isolates causing symptomatic infections. They were detected from surgical wounds (*n* = 45), urine samples (*n* = 37), hemocultures (*n* = 6), drainages (*n* = 2), stomach aspirates (*n* = 2), and ascites (*n* = 2), and all these isolates were labeled “Clinical”. The remaining 69 VRE were isolated in fecal screening of patients with high risk for colonization and were labeled “Intestinal”. A total of 23 out of the 69 intestinal VRE strains were described previously [38].

**Figure 1:**
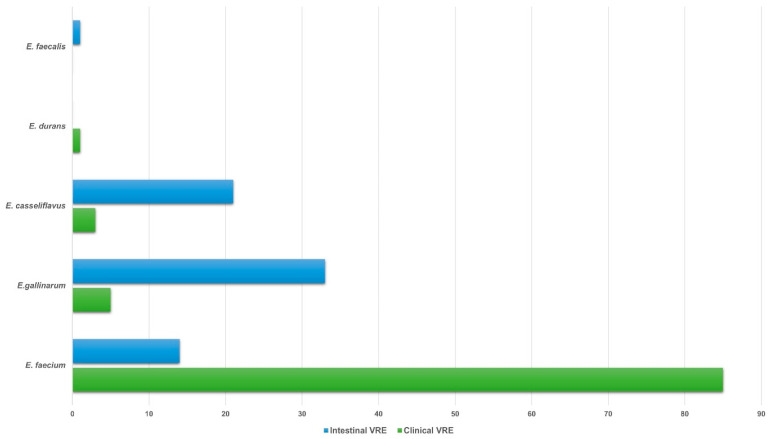
Species distribution of 94 clinical and 69 intestinal VRE isolates. Abbreviations: VRE, vancomycin-resistant enterococci.

The species distribution of VRE isolates was as follows: 85 clinical and 14 intestinal *E. faecium*, 5 clinical and 33 intestinal *E. gallinarum*, 3 clinical and 21 intestinal *E. casseliflavus*, 1 clinical *E. durans* and 1 intestinal *E. faecalis* (Figure 1).

### 2.2. Culture Media

The clinical VRE were routinely isolated in the laboratory using 5% blood agar plates (BB-NCIPD Ltd., Sofia, Bulgaria) or ChromID CPS Elite agar (bioMerieux, Lyon, France) and incubated at 37 °C for 24 h. Combination of chromogenic media chromID VRE (bioMerieux, Lyon, France), Brilliance VRE (Oxoid, Hampshire, UK), HiCrome VRE Modified (HIMEDIA, Mombaai, India) and a bile esculin azide broth (BEAV) with 6 μg/mL vancomycin (Liofilchelm, Roseto degli Abruzzi, Italy) were used for isolation of the intestinal VRE as previously described [39,40]. Briefly, the inoculated culture media were incubated at 37 °C and were observed for growth at 24 h and 48 h. Preliminary identification of VRE on each chromogenic media was based on the appropriate color of the colony. The color of positive BEAV broths turn black and were transferred on 5% BAP and chromID CPS Elite agar for an additional 24 h incubation.

The Gram-positive and catalase-negative cocci were tested with L-pyrrolidonyl arylamidase (PYR) test (Liofilchem, Italy), lucine amino peptidase (LAP) test (Oxoid, UK), ability to grow in 6.5% NaCl and bile esculin agar (Liofilchem, Italy) as well as for the presence of streptococcal D antigen. In addition, the fermentation activity to mannitol, sorbose and methyl-α-D glucopyranoside (MGP) (Oxoid, UK), the arginine hydrolysis, motility and pigment production were used for differentiation of the enterococci to group level. Species identification of the isolates was confirmed by Vitek 2 Compact system (bioMerieux, France).

### 2.3. Antimicrobial Susceptibility

Vancomycin and teicoplanin minimum inhibitory concentrations (MICs) were determined by E-test (Liofilchem, Italy) which is an agar diffusion method for direct detection of minimum inhibitory concentrations (MIC) of the antimicrobial agents. The E-test utilizes a strip (60/5 mm) that has been impregnated with exponential concentrations of the antibiotic to be studied. The strip was placed on the surface of an agar plate that has been inoculated with the tested microorganism and incubated at 35 °C for 18–24 h. During that time, the antibiotic diffused outward from the strip and an elliptical zone of growth inhibition is formed. The point of crossing the ellipse was determined as MIC. The results of the test were interpreted according to the recommendations of The European Committee on Antimicrobial Susceptibility Testing (EUCAST Breakpoint tables for interpretation of MICs and zone diameters) guidelines.

### 2.4. Amplification of Antibiotic Resistance and Virulence Genes

The genes for vancomycin resistance (*vanA*, *vanB*, *vanC*, *vanD*, *vanM*, *vanN*) and virulence factors (collagen-binding proteins (*ace*/*acm*), aggregation substance (*asa1*), enterococcal surface protein (*esp*), endocarditis-specific antigen A (*efaA*), cytolysin (*cylA*), gelatinase (*gelE*) and hyaluronidase (*hyl*) were detected by multiplex PCR using previously described primers sequences [41,42,43] and protocols [38]. A complete list of the primers with sequences is included in Table 1. Briefly, a modified PCR mix (20 μL) for detection of the investigated genes was applied. It contained a 10 ng DNA template, 0.4 μM (each) primer, 200 μM (each) dNTPs (*Canvax*, *Spain*), 1 U of Taq (*Canvax*), 2.5 mM MgCl_2_ (*Canvax*), 1X reaction buffer (*Canvax*), and ultrapure PCR H_2_O (*Canvax*).

The PCR tal conditions for the detection of *van* genes were as follows: initial denaturation (94 °C for 4 min), followed by 30 cycles of denaturation (94 °C for 30 s) and annealing (62 °C for 35 s) and extension (68 °C for 1 min), with a single final extension of 7 min at 68 °C.

The PCR amplification protocol to detect genes for virulence factors was as follows: initial denaturation (95 °C for 4 min), followed by 34 cycles of denaturation (96 °C for 20 s), annealing (53 °C for 25 s), extension (72 °C for 30 s), and final extension at 72 °C for 3 min. Capillary electrophoresis was used for the visualization and analysis of amplified PCR products.

### 2.5. Statistical Analysis

Statistical analysis was carried out in IBM SPSS Statistics for Windows, version 27 (IBM Corp., Armonk, NY, USA). The prevalence of virulence genes between clinical and intestinal *E. faecium* isolates was evaluated using Fisher’s exact test. Statistical significance was defined when *p*-value was below 0.05. The results are shown as percentages.

## 3. Results

A total of 107 (65.6%) VRE were carriers of virulence factors: 85 clinical and 14 intestinal *E. faecium* isolates, 6 intestinal *E. gallinarum*, and the single isolates *E. durans* and *E. faecalis*. Genes for virulence factors were not detected in the tested *E. casseliflavus* isolates.

### 3.1. Antimicrobial Susceptibility and Antibiotic Resistance Genes

**Figure 2:**
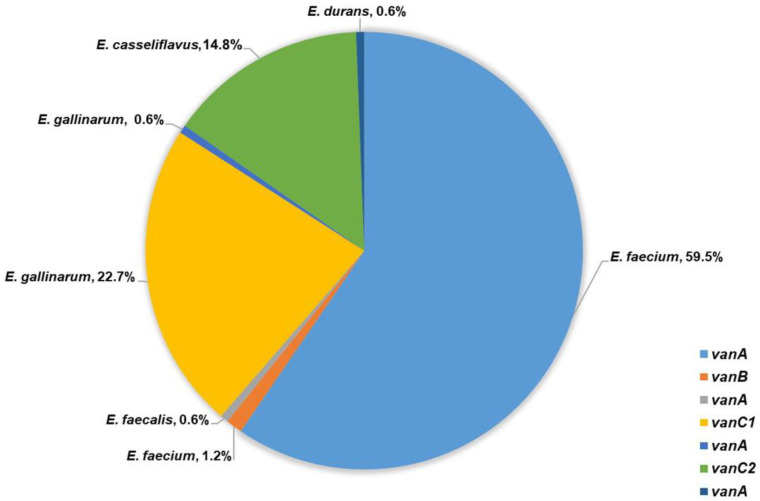
Frequency of *van* genes detected among 163 VRE.

Among the tested 99 *E. faecium* isolates, 13 intestinal and 84 clinical expressed VanA phenotype of glycopeptide resistance with high-level resistance to vancomycin (MIC ≥ 256 μg/mL) and varying resistance to teicoplanin (MICs: 6–256 μg/mL). The presence of *vanA* gene was confirmed in all of them. The other two *E. faecium* (one intestinal and one clinical) showed low-level resistance to vancomycin (MIC = 8 μg/mL), susceptibility to teicoplanin (MIC = 0.5 μg/mL) and *vanB* was detected. VanB phenotype was confirmed in the intestinal *E. faecalis* isolate with low-level resistance to vancomycin (MIC = 12 μg/mL), susceptibility to teicoplanin (MIC = 0.5 μg/mL) and *vanB* gene was confirmed. In addition, high-level resistance to vancomycin (MIC ≥ 256 μg/mL), resistance to teicoplanin (MIC = 16 μg/mL) and a presence of *vanA* gene was demonstrated by the single clinical *E. durans* isolate (Figure 2).

A total of 37 out of 38 tested *E. gallinarum* and all 24 *E. casseliflavus* showed VanC phenotype with MICs to vancomycin between 2 μg/mL and 16 μg/mL and susceptibility to teicoplanin (MIC from 0.38 μg/mL to 1.5 μg/mL). *E. gallinarum* strains were positive for *vanC1* and *E. casseliflavus* for *vanC2*. A single intestinal *E. gallinarum* isolate expressed high-level resistance to vancomycin, teicoplanin (MIC ≥ 256 μg/mL), and *vanA* gene was confirmed (Figure 2).

### 3.2. Prevalence of Virulence Genes in Clinical Isolates

Positive for virulence determinants were 91.5% (85 *E. faecium* and 1 *E. durans*) of the investigated clinical strains (Table 2). The most commonly detected genes among the isolates were *esp* (*n* = 84), *acm* (*n* = 84), and *gelE* (*n* = 5). A total of 77 out of 85 tested *E. faecium* were positive for *acm* and *esp*. Third virulence gene was confirmed in six isolates (7.1%)—three strains were positive for *hyl* and three for *gelE*. One *E. faecium* carried only *gelE* and another has four virulence genes (*gelE*, *asa1*, *esp* and *ace*). The combination of *acm* and *hyl* was confirmed in *E. durans* (Table 2). None of the tested *E. gallinarum* and *E. casseliflavus* was positive for virulence genes.

### 3.3. Prevalence of Virulence Genes in Intestinal VRE Isolates

One or more virulence determinants (*ace/acm*, *asa1*, *cylA*, *efaA*, *esp*, *gelE* and *hyl*) were detected in 30.4% intestinal isolates (14 *E. faecium*, 6 *E. gallinarum* and 1 *E. faecalis*) (Table 3). Among them, the most frequently identified genes were *esp* (*n* = 18), *acm* (*n* = 17), and *hyl* (*n* = 4). The combination of *acm* and *esp* was found in 12 (85.7%) out of 14 *E. faecium*. An additional virulence gene *hyl* was detected in two enterococci (15.3%). In *E. faecalis*, four genes (*gelE*, *asa1*, *efaA* and *ace*) were confirmed. Among the six *E. gallinarum* isolates, three (50%) were carriers of *acm* in combination with *esp* or *hyl*, two (33.3%) were positive for *hyl* or *esp*, and one (16.7%) was positive for *asa1*, *efaA*, *esp*, *ace*, and *cylA* (Table 3). Genetic determinants of virulence were not found in the tested 27 *E. gallinarum* and 21 *E. casseliflavus*.

### 3.4. Comparative Prevalence of Virulence Genes Among Clinical E. faecium and Intestinal E. faecium

**Figure 3:**
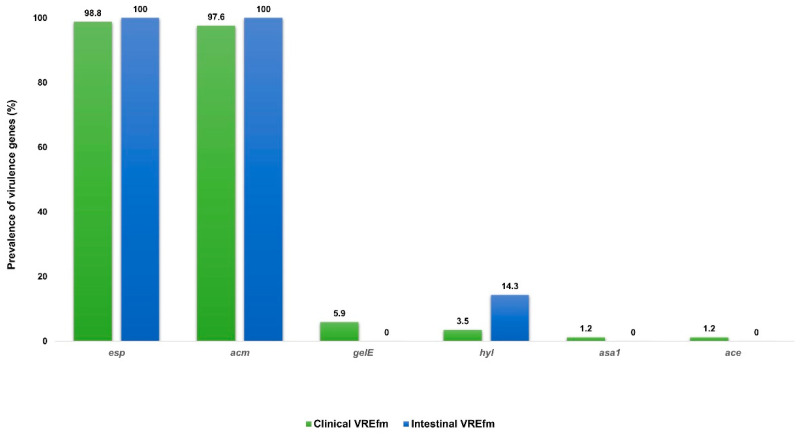
Prevalence of virulence genes among 85 clinical and 14 intestinal *E. faecium*. Abbreviations: VREfm: vancomycin-resistant *Enterococcus faecium*; *esp*, enterococcal surface protein; *acm*/*ace*, adhesion to collagen of *E. faecalis/E. faecium*; *gelE*, gelatinase; *hyl*, hyaluronidase; *asa1*, aggregation substance.

The prevalence of genes encoding virulence factors in the two studied *E. faecium* groups is presented in Figure 3. There was no statistically significant difference in the prevalence of the genes encoding virulence determinants between the clinical and intestinal *E. faecium*. The *esp* was confirmed in 98.8% clinical and 97.6% intestinal strains (*p* < 0.999), *acm*—100% vs. 100% (*p* < 0.999), *gelE*—5.9% vs. 0% (*p* < 0.999), *hyl*—3.5% vs. 14.3% (*p* < 0.1451), and *asa1* and *ace* 1.2% vs. 0% (*p* < 0.999).

## 4. Discussion

Enterococci are widely spread in the nature. They can be found in soil, water, food, and various animals. In healthy people, they are part of the normal microbiota of the small and large intestines and are less commonly found in other areas [47]. Dominant enterococcal species in the human gut are *E. faecium* and *E. faecalis*. *E. faecium* is also the main species in the gut of pigs, cattle, and poultry (production animals). In the environment, prevalent non*-faecalis*/non*-faecium* species include *E. mundtii*, *E. casseliflavus*, etc. [2].

A distinctive characteristic of enterococci is their antibiotic resistance to various agents [48], which is significantly influenced by the geographical location. According to the SENTRY Antimicrobial Surveillance Program [5], ampicillin resistance in *E. faecium* was the highest in Asia-Pacific (91.6%), followed by Europe (90.8%), North America (89.6%), and Latin America (81.6%). In all four regions, the most common glycopeptide resistance phenotypes were VanA and VanB, as VanA predominated. Its frequency varied in *E. faecium* isolates from 19.0% in Europe to 64.7% in North America. Geographical differences were also confirmed in streptomycin susceptibility among the tested enterococci [5].

In addition to antibiotic resistance in *Enterococcus*, many studies testified for the wide distribution of different genes encoding virulence factors among them. These genetic determinants play an important role in the pathogenesis of VS/VR enterococcal infection and colonization in healthy individuals. It is well known that, once colonizing the gut of hospitalized patients, VRE remain there for weeks or months [49,50]. In addition to being spread by direct patient-to-patient contact, VRE are also transmitted indirectly through transient carriage on the hands of the healthcare personnel, contaminated medical instruments, and other objectives in the hospital environment [51,52]. Enterococci survive for long period of time on various surfaces; they are tolerant to heat, chlorine, and some alcohol preparations. All these factors increased the possibility of their transmission and spreading of antimicrobial resistance and virulence genes in hospitalized patients [3,53].

This study found a high prevalence of virulence determinants in a total of 86 clinical VRE, in contrast with data from Southwest Nigeria which testified for the low occurrence of virulence factors in clinical VRE [54]. Also, the current results showed that there was no significant difference in the circulating genes encoding virulence factors. The most common combination of virulence genes was *esp* and *acm*. Two or more genetic determinants were detected in 84 *E. faecium* and in 1 *E. durans*. High prevalence of *esp* (87%) among VR *E. faecium* was also described by Cakirlar et al. [55]. Song et al. [56] studied 40 VR *E. faecium* and confirmed the presence of *esp* and *hyl* genes in 100% and 92.5% of the isolates, respectively. Research from 2023 revealed that the *gelE* gene is the most common among VR *E. faecium* (76%) and VR *E. faecalis* (69%) [57]. Bulgarian authors investigated the prevalence of genes encoding virulence factors among a total of 110 clinical vs. *E. faecium* and found *efaA* in 88.5%, *acm* in 72.8%, *hyl* in 24.2%, *asa1* in 22.8%, *gelE* in 17.1%, and *esp* in 4.3% of the isolates [58].

Usually, genes encoding aggregation substance synthesis (*asa1*), efaA protein (*efaA*), gelatinase (*gelE*), and cytolysin (*cyl*) are commonly detected in *E. faecalis* [59,60,61]. Some publications testified for the presence of these virulence determinants also in *E. faecium* [31,36,58,62]. Nasaj et al. [36] found *asa1* gene in 51 VR *E. faecium*. In another study, *gelE*, *esp*, and *asa1* were demonstrated in 45 (100%), 36 (80%), and 33 (73.3%) clinical VR *E. faecium*, respectively [62]. In the testing of 46 clinical vs. *E. faecium* for carriage of virulence determinants, *asa1* was confirmed in 9 of them, and *gelE* and *cylA* in 7 and 6 isolates, respectively [31]. In addition, the collagen-binding protein in *E. faecalis* was usually encoded by *ace* and in *E. faecium* by *acm*, although Haghi et al. [37] reported the presence of *ace* in 89.8% of VR *E. faecalis* and in 80% of VR *E. faecium*. An Iranian study found the *acm* gene in 87 (81%) *E. faecium*, and in 7 of them, it was in combination with *ace* [63]. All these data testified for the circulation of *acm* and *ace* in both *E. faecalis* and *E. faecium*.

Determinants of enterococcal pathogenicity have been described among isolates from different origins—clinical, animal, or food. However, data on their distribution among VRE isolated from the human intestinal tract are limited. The results of this study showed the presence of genes encoding virulence factors in 30.4% of a total of 69 intestinal VRE. All 14 *E. faecium* and 1 *E. faecalis* were carrying virulence genes, whereas only 6 (5 *vanC E. gallinarum* and 1 *vanA E. gallinarum*) out of 54 non-*faecalis*/non-*faecium* VRE were positive. These data testified for the higher prevalence of virulence determinants among *E. faecium* compared to *E. gallinarum* and *E. casseliflavus*. The current results were similar to those of Sienko et al. [32] who found that the number and types of virulence determinants was significantly higher among *E. faecium* strains in comparison with uncommon clinical enterococcal isolates such as *E. avium*, *E. gallinarum*, *E. casseliflavus*, and *E. durans*.

The majority of intestinal VRE (19/90.5%) in that research were carriers of two or more virulence genes. The *acm* gene was detected in 85.7% of the isolates, *esp* in 81% and *hyl* in 19% of them. Similar data was reported from Chinese authors [35] that found the *esp* in 62 (89.9%) *E. faecium* and *hyl* in 19 (27.5%). The two genes were in combination in 18 (28.6%) of the positive isolates, and in 45 (71.4%) they were presented alone. In the testing of 26 intestinal VRE, Biswas et al. [31] detected virulence determinants in 19 (61.5%), while a combination of 2 genes was only confirmed in 5 of them. The predominant genes among the positive strains were *gelE* (29.2%), *esp* (29.2%), and *asa1* (25.0%). In the study of 35 intestinal *E. faecium*, 34 (97.1%) of them were carriers of the *esp* gene, 21 (60%) of *asa1*, 18 (51.4%) of *gelE*, and only 1 isolate (2.8%) was positive for *hyl* [62].

Regarding the investigated *vanC* enterococci in the present study, *E. gallinarum* isolates (6/33) were more likely to acquire virulence determinants than *E. casseliflavus* (0/21). Two of the positive *E. gallinarum* carried one virulence gene, three strains were positive for two genes, and one isolate had five virulence determinants. These results are in contrast with Dworniczek et al. [64,65] that reported an absence of virulence factor in *E. gallinarum* and *E. casseliflavus* isolated from urinary catheters and other clinical specimens. Other authors have described the presence of *cylA*, *hyl* or *asa1* genes in single intestinal *E. gallinarum* isolates [31,66]. In 2011, Radhouani et al. [67] found a carriage of genes encoding virulence factors in seven out of eight tested *E. gallinarum* isolated from red fox faces.

The prevalence of virulence genes among the clinical *E. faecium* and intestinal *E. faecium* in that research were similar. The genes *acm* and *esp* and combination between them were the most commonly detected in the two studied groups. Six *E. gallinarum* were also positive for virulence genes, whereas none of the clinical *E. gallinarum* expressed virulence determinants. In *E. casseliflavus* isolates from both groups, genes encoding virulence factors were not confirmed. Biswas et al. [31] compared the distribution of virulence determinants in vs. and VR enterococci isolated from clinical and intestinal samples. The authors found that the frequency of virulence genes was higher among VRE compared to VSE and the prevalence of the studied genetic determinants was comparable between clinical and intestinal VRE.

The present results testified for circulation of VR *E. faecium* with almost identical virulence and *van* genes profile among both clinical and intestinal isolates. Reliable restriction measures to avoid the spread of VRE, together with measures for their early detection in infected and colonized patients could be of high importance in reduction in VRE HAIs/colonization and might prevent the distribution of additional virulence and resistance genes between these problematic microorganisms. Regardless of the small sample size and rare species detected, which is a weakness of this study, a future research goal is the genomic sequencing for PAI analysis of the VRE collection.

## 5. Conclusions

High diversity of virulence determinants was found in more than half (65.6%) of the studied VRE. A combination of two genes, mainly *acm* and *esp*, was detected in 94 (87.8%) of the positive isolates. The presence of virulence factors among intestinal *E. gallinarum* was low, while there was complete lack of such virulence genes in clinical *E. gallinarum* and clinical/intestinal *E. casseliflavus*. In comparison, in clinical and intestinal *E. faecium*, similar virulence factors were expressed and their prevalence was considerably high. Usually, the virulent enterococci are opportunistic pathogens in animals, and in rare cases, they are associated with serious infections. The animals are the main reservoir for the transmission of enterococci containing virulence and resistance genes to healthy humans. The aforementioned fact confirms that enterococci impact both animal and public health. To my knowledge, this is the first study in Bulgaria revealing the distribution of virulence genes among a significant number of VRE of clinical and especially intestinal origin.

## Figures and Tables

**Table 1:** PCR primers used in the study.

Gene	Description	Sequence (5′-3′)	Size of PCR Product (bp)	Ref.
*vanD*	VanD_F1 VanD_R2	TGGAATCACAAAATCCGGCG TWCCCGCATTTTTCACAACS	311	[41]
*vanM*	VanM_F1 VanM_R1	GGCAGAGATTGCCAACAACA AGGTAAACGAATCTGCCGCT	425	[41]
*vanC2*	VanC2_F1 VanC2_R4	GCAAACGTTGGTACCTGATG GGTGATTTTGGCGCTGATCA	523	[41]
*vanB*	VanB_F1 VanB_R1	GATGTGTCGGTAAAATCCGC CCACTTCGCCGACAATCAAA	640	[41]
*vanA*	VanA_F1 VanA_R1	GCAAGTCAGGTGAAGATGGA GCTAATACGATCAAGCGGTC	721	[41]
*vanC1*	VanC1 _5 VanC1_6	GTATCAAGGAAACCTCGCGA CGTAGGATAACCCGACTTCC	836	[41]
*vanN*	VanN_F1 VanN_R1	CCTCAAATCAGCAGCTAGTG GCTCCTGATAAGTGATACCC	941	[41]
*gelE*	Gelatinase	TATGACAATGCTTTTTGGGAT AGATGCACCCGAAATAATATA	213	[44]
*hyl*	Hyaluronidase	ACAGAAGAGCTGCAGGAAATG GACTGACGTCCAAGTTTCCAA	276	[44]
*asa1*	Aggregation substance	CACGCTATTACGAACTATGA TAAGAAAGAACATCACCACGA	375	[44]
*efaA*	*E. faecalis* antigen A	CGTGAGAAAGAAATGGAGGA CTACTAACACGTCACGAATG	499	[45]
*esp*	Enterococcal surface protein	AGATTTCATCTTTGATTCTTGG AATTGATTCTTTAGCATCTGG	510	[44]
*acm*	Adhesion to collagen of *E. faecium*	GGCCAGAAACGTAACCGATA CGCTGGGGAAATCTTGTAAA	353	[43]
*ace*	Adhesion to collagen of *E. faecalis*	GGAATGACCGAGAACGATGGC GCTTGATGTTGGCCTGCTTCCG	616	[46]
*cylA*	Cytolysin	ACTCGGGGATTGATAGGC GCTGCTAAAGCTGCGCTT	688	[44]

**Table 2:** Profile of virulence genes in 86 clinical VRE.

Species	Number of the Isolates	Virulence Gene Profile
*E. faecium*	77	*esp*, *acm*
*E. faecium*	3	*hyl*, *esp*, *acm*
*E. faecium*	3	*gelE*, *esp*, *acm*
*E. faecium*	1	*gelE*
*E. faecium*	1	*gelE*, *asa1*, *esp*, *ace*
*E. durans*	1	*hyl*, *acm*

Abbreviations: VRE, vancomycin-resistant enterococci; *gelE*, gelatinase; *hyl*, hyaluronidase; *asa1*, aggregation substance; *efaA*, *E. fae*ococcal surface protein; *cylA*, cytolyion to collagen of *E. faecalis*; *acm*, adhesion to collagen of *E. faecium*.

**Table 3:** Profiles of virulence gene in 21 intestinal VRE isolates.

Species	Number of the Isolates	Virulence Gene Profile
*E. faecium*	12	*esp*, *acm*
*E. faecium*	2	*hyl*, *esp*, *acm*
*E. gallinarum*	2	*esp*, *acm*
*E. gallinarum*	1	*hyl*, *acm*
*E. gallinarum*	1	*asa1*, *efaA*, *esp*, *cylA*, *ace*
*E. gallinarum*	1	*hyl*
*E. gallinarum*	1	*esp*
*E. faecalis*	1	*gelE*, *asa1*, *efaA*, *ace*

Abbreviations: VRE, vancomycin-resistant enterococci. *gelE*, gelatinase; *hyl*, hyaluronidase; *asa1*, aggregation substance; *efaA*, *E. faecalis* antigen A; *esp*, enterococcal surface protein; *cylA*, cytolysin; *ace*, adhesion to collagen of *E. faecalis*; *acm*, adhesion to collagen of *E. faecium*.

## Data Availability

The original contributions presented in this study are included in the article. Further inquiries can be directed to the corresponding author.
